# Supplementary material for: CIRCUST: A novel methodology for temporal order reconstruction of molecular rhythms; validation and application towards a daily rhythm gene expression atlas in humans
Source: PLoS Comput Biol. 2023 Sep 28;19(9):e1011510. doi: 10.1371/journal.pcbi.1011510 (PMC10564179; doi:10.1371/journal.pcbi.1011510)
Supplement: S1 Text — Supplementary material for this paper including figures, tables, additional methodological details, simulations, and CIRCUST comparisons with other methods. (PDF) [file pcbi.1011510.s001.pdf]

Supporting Information for "CIRCUST: a novel methodology for temporal order reconstruction of molecular rhythms; validation and application towards a daily rhythm gene expression atlas in humans"

Yolanda Larriba[1,2], Ivy C. Mason[3,4], Richa Saxena[4,5,6,7], Frank A.J.L. Scheer[3,4,8], and Cristina Rueda[1,2]

[1]Department of Statistics and Operational Research, University of Valladolid, Spain.

[2]Mathematics Research Institute of the University of Valladolid, University of Valladolid, Spain.

[3]Medical Chronobiology Program, Division of Sleep and Circadian Disorders, Departments of Medicine and Neurology, Brigham and Women's Hospital, Boston, Massachusetts, United States of America.

[4]Division of Sleep Medicine, Harvard Medical School, Boston, Massachusetts, United States of America.

[5]Center for Genomic Medicine and Department of Anesthesia, Critical Care and Pain Medicine, Massachusetts General Hospital, Boston, Massachusetts, United States of America.

[6]Division of Anesthesia, Harvard Medical School, Boston, Massachusetts, United States of America.

[7]Program in Medical and Population Genetics, Broad Institute of Massachusetts Institute of Technology and Harvard, Cambridge, Massachusetts, United States of America.

[8]Broad Institute of Massachusetts Institute of Technology and Harvard, Cambridge, Massachusetts, United States of America.

Contents

1 Supplementary Figs **3**

2 Supplementary Tables **10**

3 Supplementary text: Advanced Methodological details **14**

3.1 FMM model approach . . . 14

3.2 CPCA Temporal order estimation. Starting point and direction choice . . 15

3.3 CPCA Outliers sample detection . . . 17

3.4 Seed gene selection and circular ordering . . . 17

3.5 R^2−based goodness of fit criteria . . . 17

4 Supplementary results: Simulation **19**

5 CIRCUST comparison against CYCLOPS and CHIRAL **20**

5.1 CIRCUST against CYCLOPS . . . 20

5.2 CIRCUST against CHIRAL . . . 21

6 List of acronyms **24**

This supplementary material appends several issues mentioned in the manuscript. First, supplemental Figs and Tables are provided in order to accomplish the analyses given in the main text. Next, two supplemental texts are included to expand the methods and results sections of the main text. Finally, we provide a list of the acronyms used in this work.

1 Supplementary Figs

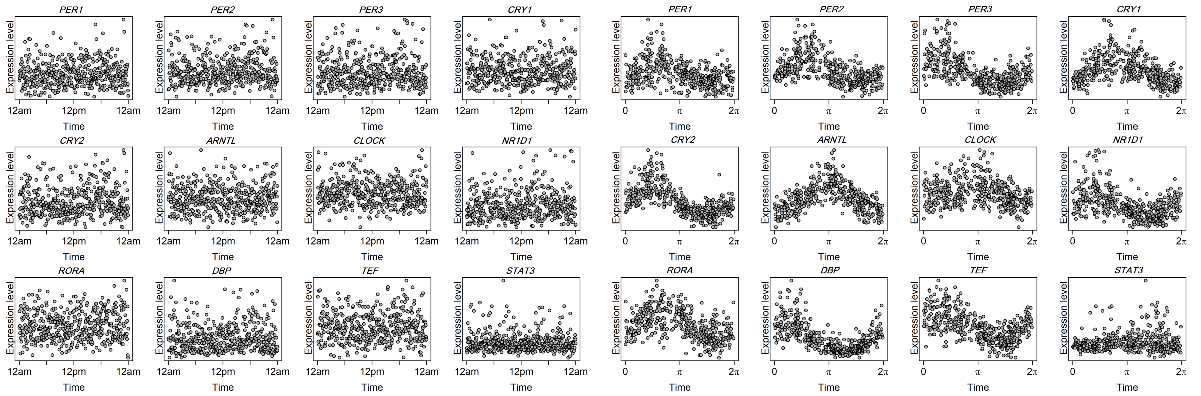

Figure A: Core clock gene expression patterns from Skin sun-exposed (Lower leg) from GTEx dataset. Left: gene expressions as a function of TOD times. Right: gene expressions as a function of CIRCUST estimated times ($[0, 2\pi)$).

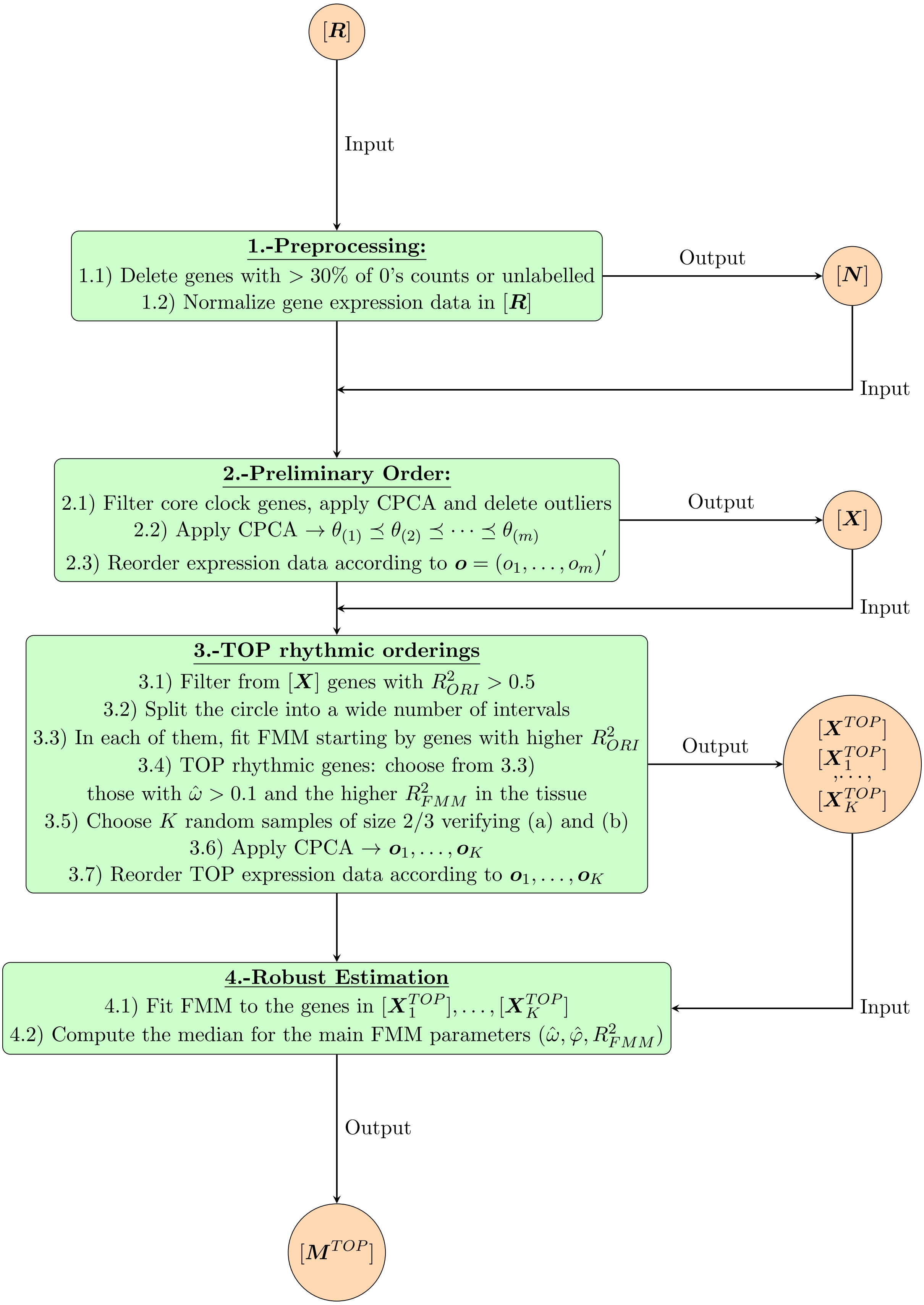

Figure B: Outline of the CIRCUST methodology.

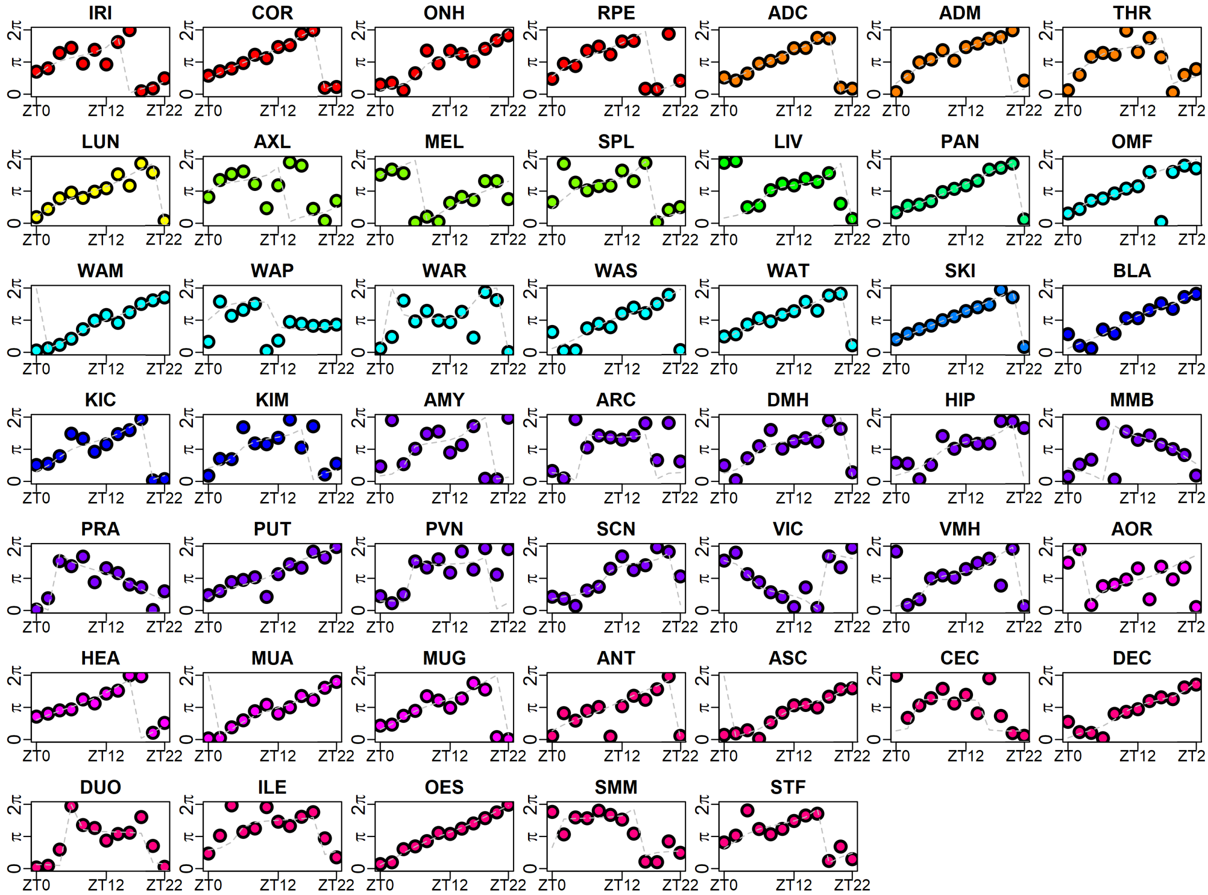

Figure C: Circular-circular regression model for the real clock times (X-axis) and CIRCUST$_{prior}$ estimated times (Y-axis) across the 47 baboons' tissues selected. Horizontal axis: sampling real clock times along 24-h (ZT0,ZT2,...,ZT22). Vertical axis: CIRCUST estimated times in $[0, 2\pi)$. Time 0-h is the same as 24-h and the phase 0 is the same as 2π. The diagonal line observed for most of the tissues is used as a marker of the coherence between the orders. Colors match with the 12 functional organs groups considered in GSE98965. See Table A for tissue names.

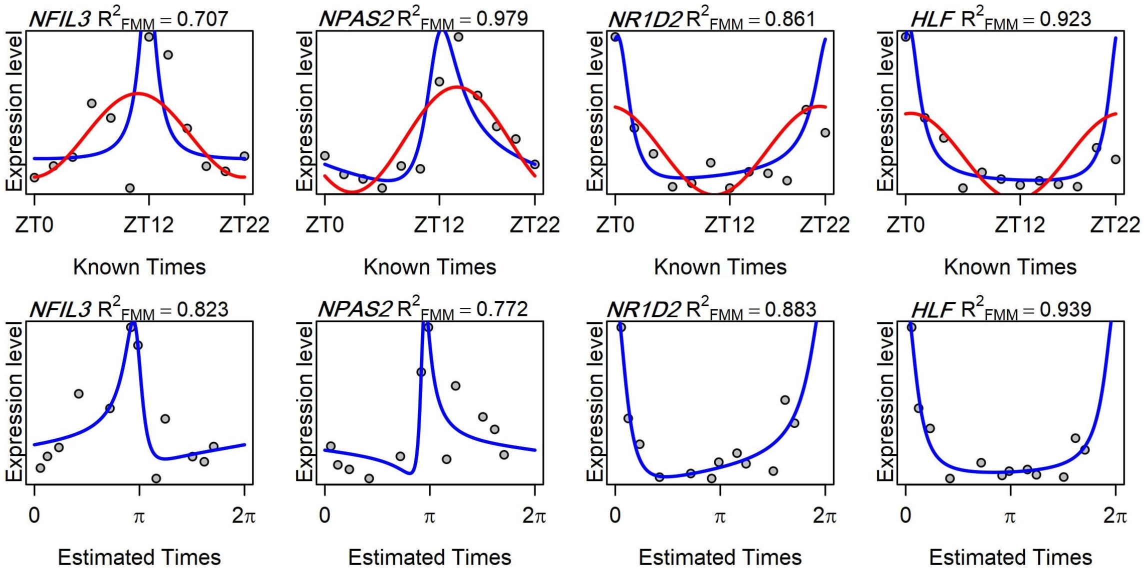

Figure D: Expression of selected clock genes *NFIL3*, *NPAS2*, *NR1D2* and *HLF* in White Adipose Mesenteric (WAM) tissue from baboons (GSE98965). Top panels: expressions as function of known times ZT0, ZT2,...,ZT22. Bottom panels: expressions as function of CIRCUST estimated times. FMM predictions are shown as blue solid lines. Cosinor predictions are shown as red solid lines.

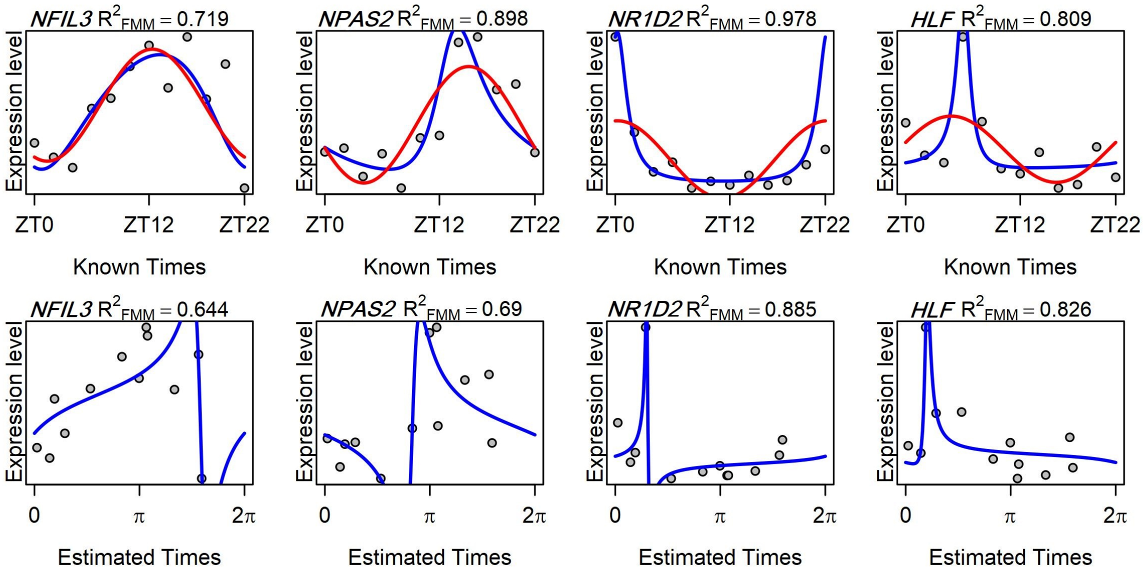

Figure E: Expression of selected clock genes *NFIL3*, *NPAS2*, *NR1D2* and *HLF* in Ascending Colon (ASC) tissue from baboons (GSE98965). Top panels: expressions as function of known times ZT0, ZT2,...,ZT22. Bottom panels: expressions as function of CIRCUST estimated times. FMM predictions are shown as blue solid lines. Cosinor predictions are shown as red solid lines.

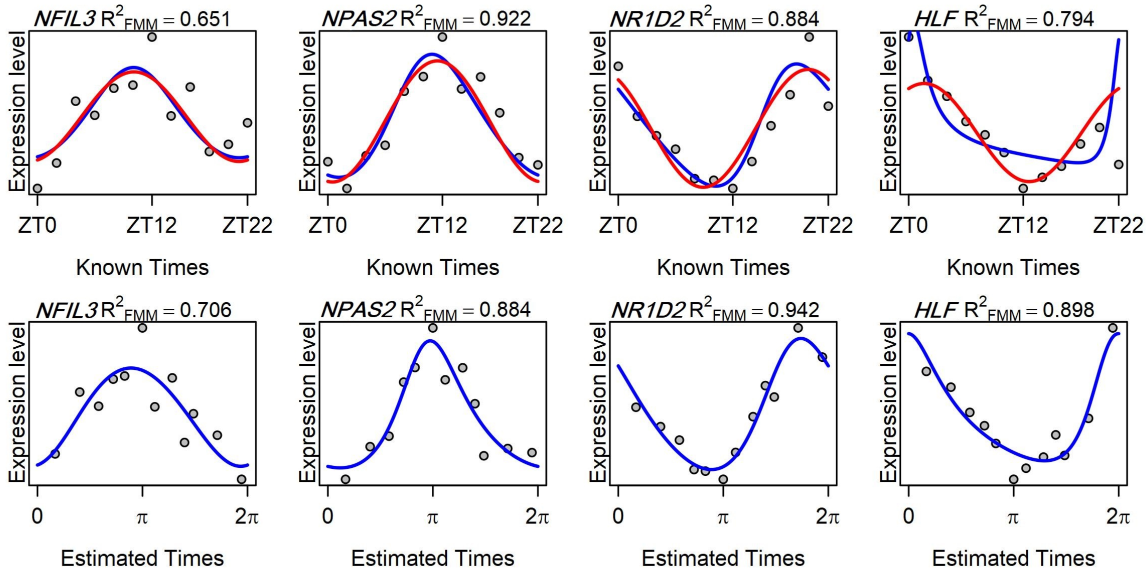

Figure F: Expression of selected clock genes *NFIL3*, *NPAS2*, *NR1D2* and *HLF* in Skin (SKI) tissue from baboons (GSE98965). Top panels: expressions as function of known times ZT0, ZT2,...,ZT22. Bottom panels: expressions as function of CIRCUST estimated times. FMM predictions are shown as blue solid lines. Cosinor predictions are shown as red solid lines.

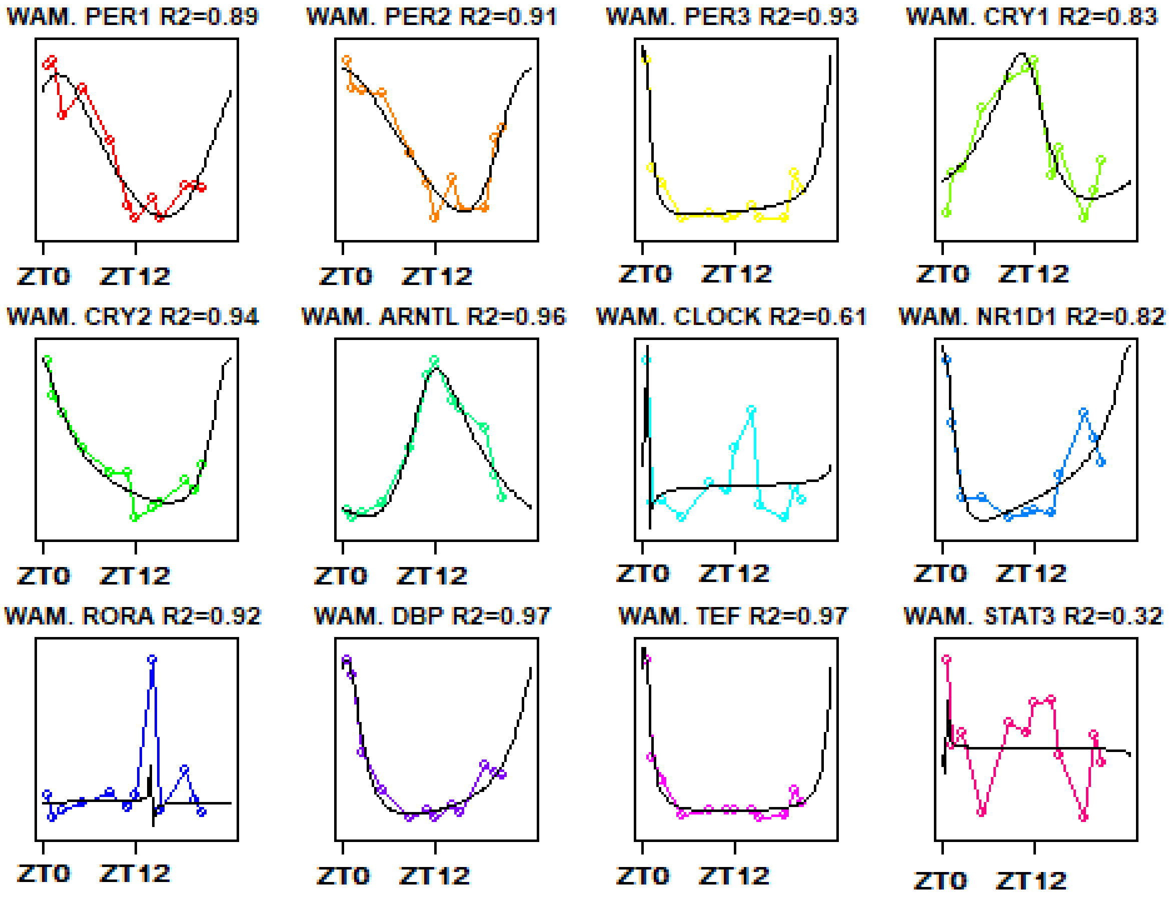

Figure G: Gene expression of seed genes in White Adipose Mesenteric (WAM) tissue from baboons (GSE98965) after discarding outliers. In Fig D, ZT0 may seem to be outlier looking at *HLF* and *NR1D2*. CIRCUST does not delete ZT0 because it is not outlier for the seed genes *PER1, PER2, CRY1, CRY2* or *ARNTL*. FMM predictions are shown as solid lines.

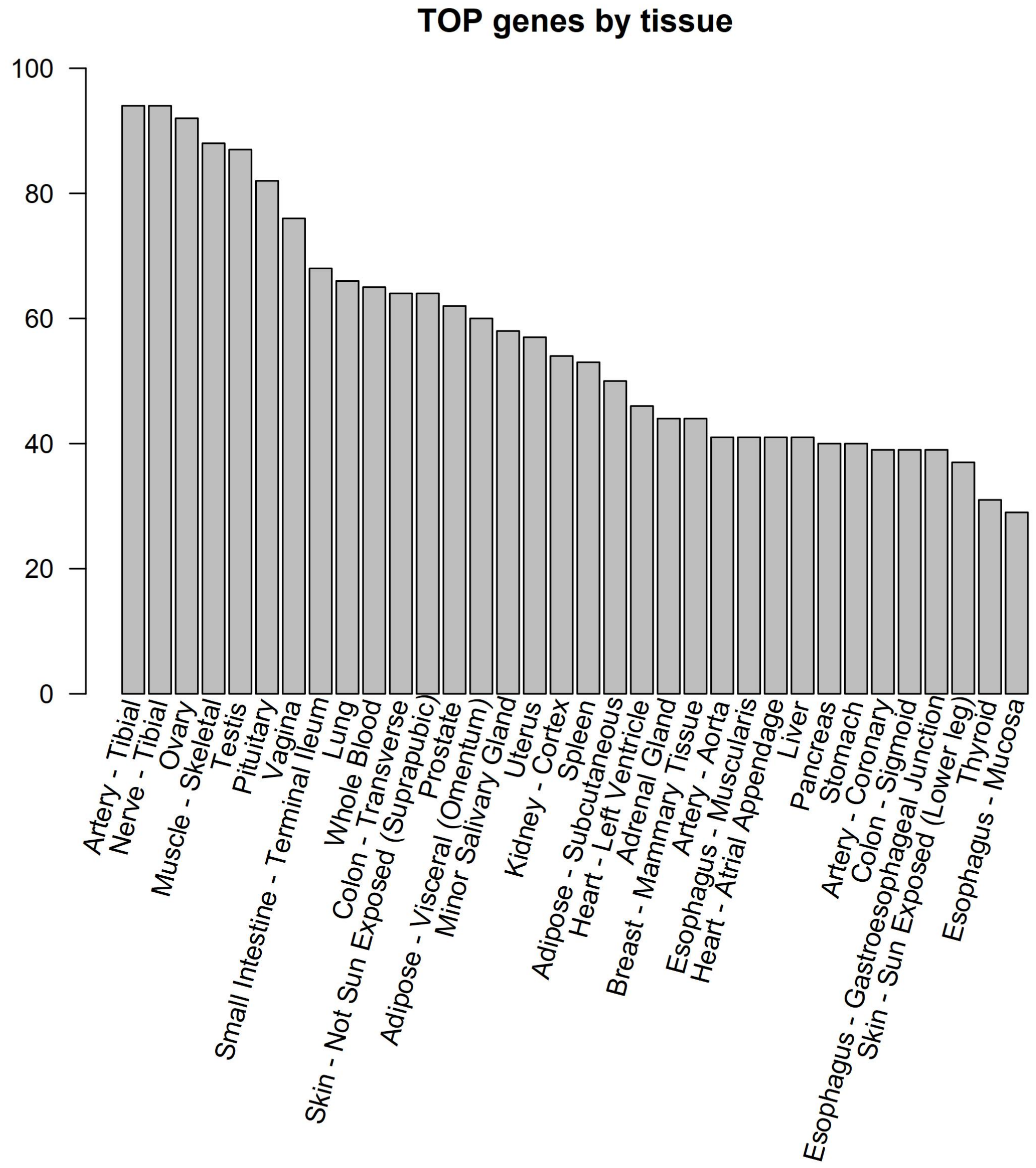

Figure H: Number of TOP rhythmic genes across the GTEx. Tissues are shown in decreasing the number of TOP rhythmic genes.

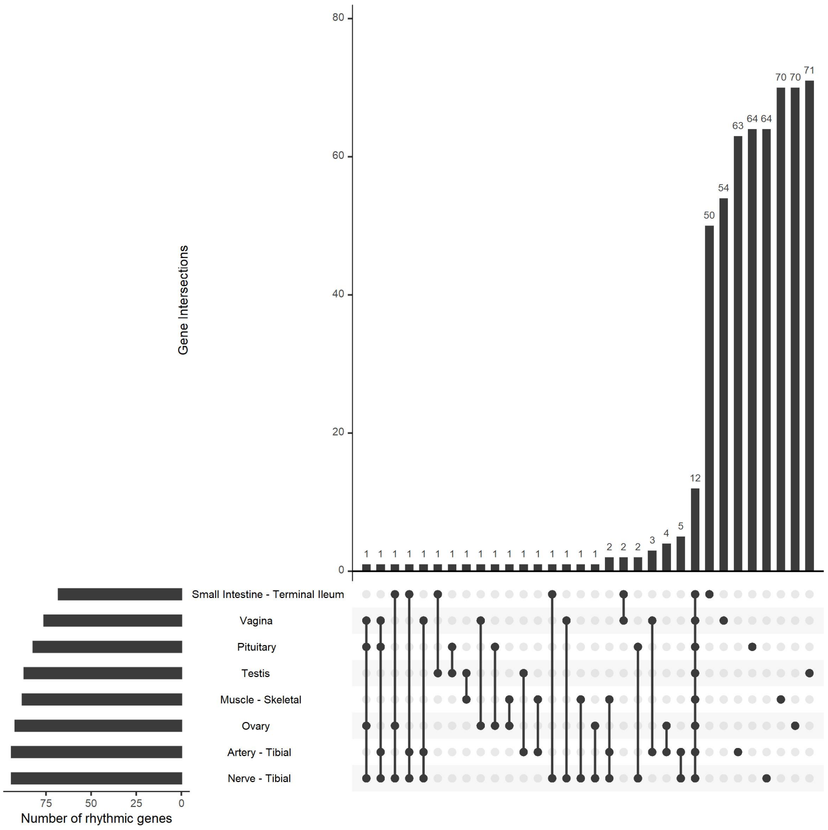

Figure I: UpSetR [1] technique to visualize TOP rhythmic gene intersections across the 34 tissues. Dots denote single tissue intersections. Bars match tissues with common TOP rhythmic genes. For better illustration, only the eight tissues with the higher number of TOP genes have been included in the analysis.

2 Supplementary Tables

Table A: Baboons tissue characterization. First column: Tissue abbreviation. Second column: Tissue name. Third column: R^2_{Ave}, average rhythmicity measure R^2_{FMM} for the 12 core clock genes as a function of estimated times. Fourth column: ρ goodness of fit measure fit measure for circular-circular regression between the real times (ZT0,ZT2,...,ZT22) and the CIRCUST estimated times ($[0, 2\pi)$). Tissues are restricted to those with $R^2_{Ave} > 0.7$ to guarantee the consistency of the results. Lines separate functional groups.

Abbreviation	Tissue	R^2_{Ave}	ρ
IRI	Iris	0.811	0.800
COR	Cornea	0.846	0.958
ONH	Optic Nerve Head	0.807	0.874
RPE	Retina Pigment Epithelium	0.740	0.871
ADC	Adrenal Cortex	0.787	0.947
ADM	Adrenal Medulla	0.799	0.899
THR	Thyroid	0.810	0.723
LUN	Lung	0.803	0.911
AXL	Axilliary Lymphonodes	0.724	0.615
MEL	Mesenteric Lymphonodes	0.746	0.833
SPL	Spleen	0.703	0.741
LIV	Liver	0.730	0.777
PAN	Pancreas	0.812	0.960
OMF	Omental Fat	0.796	0.892
WAM	White Adipose Mesenteric	0.814	0.964
WAP	White Adipose Mesenteric	0.745	0.609
WAR	White Adipose Perirenal	0.813	0.708
WAS	White Adipose Subcutaneous	0.811	0.878
WAT	White Adipose Tissue	0.892	0.961
SKI	Skin	0.788	0.953
BLA	Bladder	0.745	0.880
KIC	Kidney Cortex	0.811	0.765
KIM	Kidney Medulla	0.764	0.728
AMY	Amygdala	0.702	0.686
ARC	Arcuate Nucleus	0.745	0.680
DMH	Dorsomedial Hypothalamus	0.703	0.728
HIP	Hippocampus	0.704	0.734
MMB	Mammilary Bodies	0.735	0.789
PRA	Preoptic Area	0.702	0.741
PUT	Putamen	0.784	0.813
PVN	Paraventricular Nuclei	0.728	0.744
SCN	Suprachiasmatic Nuclei	0.711	0.729
VIC	Visual Cortex	0.740	0.706
VMH	Ventromedial Hypothalamus	0.776	0.806
AOR	Aorta	0.742	0.660
HEA	Heart	0.841	0.935
MUA	Muscle Abdominal	0.799	0.953
MUG	Muscle Gastrocnemian	0.701	0.884
ANT	Antrum	0.835	0.740
ASC	Ascending Colon	0.746	0.959
CEC	Cecum	0.701	0.734
DEC	Descending Colon	0.739	0.846
DUO	Duodenum	0.816	0.747
ILE	Ileon	0.709	0.726
OES	Oesophagus	0.903	0.973
SMM	Smooth Muscle	0.741	0.674
STF	Stomach Fundus	0.824	0.734

Table B: GTEx donor distribution by sex, age, and cause of death. Death was classified as follows. Fast: death due to accident, blunt force trauma, or suicide; Intermediate: patients who were ill but death was unexpected; Slow: death after a long illness; Sudden-Natural: fast death of natural causes, sudden unexpected deaths; Ventilator: all cases on a ventilator immediately before death.

**Sex**	Male	Female				
	65.86%	34.14%				
**Age**	20-29	30-39	40-49	50-59	60-69	70-79
	8.05%	7.25%	15.94%	32.85%	32.69%	3.22%
**Death**	Fast	Intermediate	Slow	Sudden-Natural	Ventilator	
	4.35%	4.99%	12.56%	26.73%	50.72%	

Table C: R^2_{FMM} for the time course expression of the 12 core clock genes as a function of CIRCUST times across the 34 GTEx tissues analyzed. Only the peaks of the core clock genes with $R^2_{FMM} > 0.3$ are shown in Fig 9.

	PER1	*PER2*	*PER3*	*CRY1*	*CRY2*	*ARNTL*	*CLOCK*	*NR1D1*	*RORA*	*DBP*	*TEF*	*STAT3*
Adipose - Subcutaneous	0.184	0.466	0.504	0.317	0.254	0.393	0.31	0.231	0.52	0.586	0.178	0.353
Adipose - Visceral (Omentum)	0.273	0.217	0.372	0.571	0.191	0.088	0.34	0.359	0.508	0.455	0.397	0.671
Adrenal Gland	0.109	0.074	0.531	0.308	0.176	0.366	0.559	0.396	0.34	0.714	0.661	0.446
Artery - Aorta	0.36	0.276	0.613	0.191	0.636	0.499	0.25	0.316	0.19	0.527	0.559	0.147
Artery - Coronary	0.155	0.36	0.666	0.365	0.491	0.565	0.496	0.229	0.514	0.568	0.526	0.339
Artery - Tibial	0.338	0.281	0.424	0.039	0.379	0.488	0.306	0.046	0.176	0.359	0.396	0.178
Breast - Mammary Tissue	0.405	0.54	0.687	0.445	0.419	0.637	0.241	0.517	0.613	0.61	0.324	0.22
Colon - Sigmoid	0.215	0.294	0.678	0.23	0.449	0.545	0.585	0.204	0.426	0.484	0.598	0.235
Colon - Transverse	0.535	0.077	0.592	0.517	0.652	0.367	0.671	0.377	0.75	0.332	0.422	0.299
Esophagus - Gastroesophageal	0.144	0.258	0.657	0.439	0.537	0.531	0.567	0.175	0.497	0.454	0.668	0.214
Esophagus - Mucosa	0.253	0.198	0.682	0.338	0.323	0.605	0.327	0.271	0.285	0.513	0.713	0.097
Esophagus - Muscularis	0.167	0.295	0.718	0.385	0.452	0.553	0.503	0.253	0.568	0.504	0.661	0.18
Heart - Atrial Appendage	0.338	0.306	0.64	0.437	0.373	0.342	0.303	0.211	0.48	0.451	0.668	0.345
Heart - Left Ventricle	0.222	0.104	0.692	0.557	0.481	0.564	0.714	0.227	0.376	0.471	0.758	0.341
Kidney - Cortex	0.478	0.822	0.754	0.75	0.723	0.75	0.8	0.395	0.675	0.794	0.767	0.877
Liver	0.54	0.232	0.485	0.239	0.559	0.156	0.561	0.316	0.555	0.497	0.142	0.613
Lung	0.358	0.269	0.236	0.559	0.456	0.203	0.03	0.278	0.442	0.301	0.549	0.477
Minor Salivary Gland	0.343	0.181	0.475	0.182	0.487	0.434	0.511	0.43	0.384	0.341	0.585	0.144
Muscle - Skeletal	0.442	0.302	0.451	0.338	0.409	0.557	0.559	0.166	0.507	0.42	0.287	0.199
Nerve - Tibial	0.157	0.451	0.583	0.311	0.226	0.368	0.299	0.155	0.399	0.6	0.255	0.213
Ovary	0.559	0.174	0.514	0.29	0.483	0.157	0.126	0.304	0.341	0.587	0.454	0.396
Pancreas	0.11	0.464	0.681	0.551	0.395	0.605	0.418	0.134	0.491	0.504	0.675	0.484
Pituitary	0.568	0.434	0.703	0.207	0.511	0.178	0.282	0.114	0.071	0.59	0.629	0.41
Prostate	0.101	0.167	0.491	0.309	0.286	0.283	0.49	0.22	0.516	0.473	0.472	0.433
Skin - Not Sun Exposed	0.415	0.235	0.519	0.182	0.506	0.471	0.175	0.46	0.547	0.564	0.176	0.133
Skin - Sun Exposed	0.193	0.313	0.543	0.251	0.509	0.503	0.216	0.308	0.402	0.605	0.463	0.05
Small Intestine - Terminal Ile	0.551	0.331	0.453	0.499	0.678	0.265	0.379	0.404	0.468	0.12	0.227	0.221
Spleen	0.354	0.338	0.351	0.334	0.458	0.46	0.641	0.309	0.495	0.108	0.393	0.587
Stomach	0.513	0.112	0.607	0.415	0.576	0.543	0.545	0.216	0.583	0.635	0.697	0.582
Testis	0.196	0.246	0.555	0.655	0.119	0.473	0.752	0.315	0.295	0.382	0.314	0.522
Thyroid	0.275	0.535	0.708	0.351	0.532	0.54	0.403	0.198	0.469	0.537	0.664	0.176
Uterus	0.235	0.349	0.456	0.341	0.594	0.313	0.196	0.457	0.561	0.559	0.443	0.432
Vagina	0.306	0.365	0.537	0.436	0.6	0.296	0.205	0.481	0.384	0.544	0.363	0.164
Whole Blood	0.499	0.593	0.556	0.673	0.753	0.693	0.721	0.597	0.682	0.462	0.488	0.509

Table D: Estimated t_U for the time course expression of the 12 core clock genes as a function of CIRCUST times across the 34 GTEx tissues analyzed. Only the peaks of the core clock genes with $R^2_{FMM} > 0.3$ are shown in Fig 9.

	PER1	*PER2*	*PER3*	*CRY1*	*CRY2*	*ARNTL*	*CLOCK*	*NR1D1*	*RORA*	*DBP*	*TEF*	*STAT3*
Adipose - Subcutaneous	1.196	0.865	0.173	1.417	0.397	3.142	2.196	0.306	1.395	6.073	0.093	2.031
Adipose - Visceral (Omentum)	2.855	4.046	1.941	3.491	2.195	3.142	3.484	2.127	3.082	1.855	1.927	3.788
Adrenal Gland	5.408	0.95	0.806	2.683	3.553	3.142	2.214	0.627	1.519	0.533	0.805	3.105
Artery - Aorta	1.089	0.991	0.666	3.005	1.153	3.142	3.328	1.141	3.049	0.688	0.289	3.341
Artery - Coronary	1.581	1.888	0.786	2.421	1.607	3.142	2.823	1.207	2.742	0.67	0.768	2.645
Artery - Tibial	2.124	1.719	1.352	4.382	1.717	3.142	2.795	1.971	2.637	0.549	1.912	4.578
Breast - Mammary Tissue	0.755	0.557	0.194	1.444	0.335	3.142	2.137	1.072	2.159	0.355	0.137	1.66
Colon - Sigmoid	1.412	0.801	0.889	3.654	0.796	3.142	3.01	0.786	2.149	0.497	1.038	3.897
Colon - Transverse	2.655	2.429	2.86	3.096	2.819	3.142	3.164	2.876	3.014	2.818	2.896	2.802
Esophagus - Gastroesophageal	1.332	0.831	0.82	3	0.964	3.142	2.388	0.821	1.758	0.458	0.814	2.549
Esophagus - Mucosa	1.77	1.546	0.804	3.521	1.298	3.142	2.919	1.271	2.125	0.881	0.867	4.629
Esophagus - Muscularis	1.8	1.245	1.031	3.291	1.188	3.142	2.729	0.48	2.322	0.547	0.869	3.574
Heart - Atrial Appendage	2.261	1.907	1.207	3.01	2.229	3.142	2.342	2.206	2.522	1.002	1.05	3.113
Heart - Left Ventricle	0.213	0.898	0.892	2.993	1.23	3.142	2.512	0.142	2.172	0.602	1.064	3.301
Kidney - Cortex	2.697	2.266	2.145	2.485	2.288	3.142	2.431	2.587	2.519	1.83	2.21	2.649
Liver	2.695	2.154	2.181	3.868	2.2	3.142	4.369	2.511	3.338	2.141	2.296	4.696
Lung	2.123	2.763	1.928	3.005	2.547	3.142	2.525	2.382	2.922	1.809	1.325	4.007
Minor Salivary Gland	0.139	5.065	0.749	2.589	0.252	3.142	2.474	0.385	1.724	6.124	0.688	3.274
Muscle - Skeletal	2.12	1.82	1.811	2.763	1.941	3.142	2.903	2.113	2.499	0.599	1.645	2.629
Nerve - Tibial	1.368	1.591	1.036	2.437	1.473	3.142	2.199	5.588	1.967	0.616	0.87	2.718
Ovary	3.567	3.134	2.382	4.93	3.509	3.142	5.777	2.664	3.53	1.925	2.225	4.995
Pancreas	2.552	1.822	1.022	3.1	2.144	3.142	2.243	1.544	2.573	0.736	1.403	2.743
Pituitary	0.37	1.039	1.346	5.416	0.338	3.142	2.634	0.591	3.058	1.379	1.522	5.317
Prostate	2.952	3.8	2.106	4.058	2.097	3.142	3.158	2.435	2.932	1.832	1.861	4.192
Skin - Not Sun Exposed	2.193	3.29	1.069	2.38	1.882	3.142	1.258	2.115	2.57	1.409	1.305	5.077
Skin - Sun Exposed	1.621	1.742	1.09	2.284	1.458	3.142	2.927	1.202	1.929	0.73	0.811	3.645
Small Intestine - Terminal Ileum	2.973	2.884	2.611	2.815	2.924	3.142	2.9	1.762	2.961	1.652	2.631	2.759
Spleen	2.786	2.771	2.403	2.68	3.061	3.142	2.971	2.24	2.999	3.231	2.367	2.827
Stomach	2.935	4.583	1.964	3.745	2.645	3.142	3.277	1.922	2.994	1.643	2.221	3.559
Testis	2.917	3.859	2.682	4.261	3.673	3.142	4.044	2.451	3.072	2.248	2.89	2.466
Thyroid	0.914	0.978	1.012	2.768	1.118	3.142	2.557	1.216	2.641	0.829	0.915	2.223
Uterus	2.54	1.638	1.361	2.807	1.861	3.142	3.885	2.093	2.83	1.284	1.288	3.368
Vagina	3.012	0.851	1.078	1.707	1.649	3.142	5.757	1.712	1.535	0.961	1.439	2.845
Whole Blood	1.687	2.143	2.255	2.017	1.998	3.142	2.817	1.778	1.962	1.496	2.74	3.146

3 Supplementary text: Advanced Methodological details

3.1 FMM model approach

The Frequency Modulated Möbius (FMM) proposed in [2] is a multi-purpose approach that combines a physically meaningful formulation with excellent statistical and computational properties. It is formulated as a signal plus error model where the signal is described parametrically as an oscillatory signal. The parametric formulation facilitates the interpretability and the derivation of essential elements in the analysis of oscillations. Among the distinguishing features of the FMM are: the formulation in terms of the phase (φ), which is an angular variable representing the periodic oscillation movement that efficiently allows capturing the underlying rhythmic patterns being, as well as its low influence of outlier samples (see Fig J). All the methodological details that justify the mathematical formulation of the FMM models are given in [2].

Let assume that the time points are in $[0, 2\pi)$. In any other case, transform the time points $t' \in [t_0, T + t_0]$ by $t = \frac{(t'-t_0)2\pi}{T}$. The FMM signal plus error model is defined as follows.

Definition 1. FMM model.

For the observations $t_1 < \ldots < t_m$,

$$X(t_i) = \mu(t_i) + e(t_i) = M + A\cos(\varphi(t_i)) + e(t_i); \quad i = 1, \ldots, m$$

- $M \in \mathcal{R}, A \in \mathcal{R}^+$.
- $\varphi(t) = \beta + 2\arctan(\omega \tan(\frac{t-\alpha}{2})); \alpha, \beta \in [0, 2\pi), \omega \in [0, 1]$.
- $(e(t_1), \ldots, e(t_m))' \sim N_m(0, \sigma^2)$.

The FMM parameters characterize various aspects of a rhythmic pattern. The parameter M is an intercept and A measures the signal's amplitude. α is a phase location parameter, while ω and β are parameters that describe the shape. Specifically, ω measures the sharpness, and β measures skewness and indicates upward and/or downward peak direction. Note that a sinusoidal curve corresponds to $\omega = 1$.

Other important parameters of practical use are peak and trough times, denoted by t_U and t_L, respectively, which are defined as follows:

$$t_U = \alpha + 2\arctan(\frac{1}{\omega}\tan(\frac{-\beta}{2}))$$

$$t_L = \alpha + 2\arctan(\frac{1}{\omega}\tan(\frac{\pi-\beta}{2}))$$

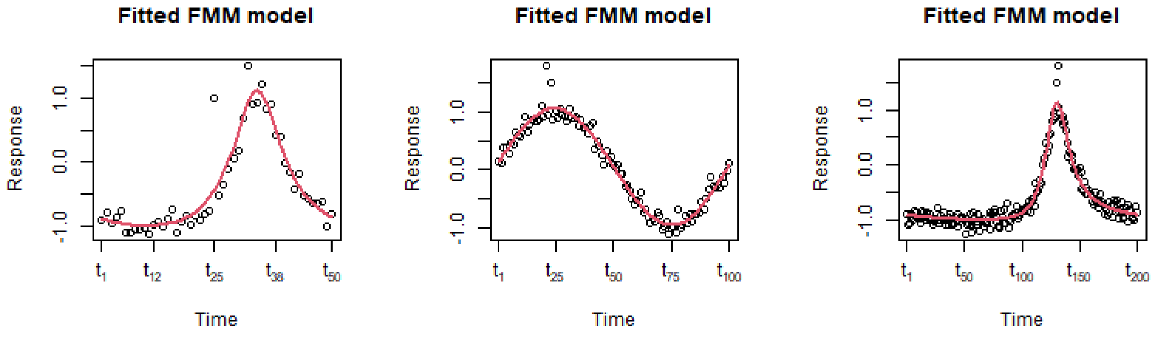

Figure J: FMM robustness against outliers samples. Simulated data from **FMM** package [3] covering different patterns and with 50 (left), 100 (middle) and 200 (right) observations.

3.2 CPCA Temporal order estimation. Starting point and direction choice

The solution of the temporal order estimation problem proposed in this work not only solves the mathematical problem of identifying a circular order, but also states the starting point and the clockwise or counterclockwise direction. Methodological details for these purposes are given below.

Let $[\boldsymbol{X}]$ be a gene expression matrix, and let $\boldsymbol{E}_1$ and $\boldsymbol{E}_2$ be the two first *eigengenes* of $[\boldsymbol{X}]$ so that the first two eigengenes account for at least 40% of the variability in a balanced manner, that is the second eigengene accounts for at least 10% of the variability. Eigengenes are linear combination of the gene expressions along the direction of the most variation in the data [4]. Under rhythmicity, the mapping of $\boldsymbol{E}_1$ against $\boldsymbol{E}_2$ reveals an underlying circular structure, see panels (a), (b) and (c) in Fig K.

The Circular Principal Component Analysis (CPCA) [5] is defined as the transformation in which the eigengenes are projected onto the unit circle as follows:

$$(e_{1,i}, e_{2,i}) = (\frac{E_{1,i}}{\sqrt{E_{1,i}^2 + E_{2,i}^2}}, \frac{E_{2,i}}{\sqrt{E_{1,i}^2 + E_{2,i}^2}}), i = 1, \dots, m$$

From these projections, CPCA computes $\boldsymbol{\theta} = (\theta_1, \dots, \theta_m)'$, the vector of angular phases that represents the temporal position of samples onto the unit circle ($[0, 2\pi)$), where $\theta_i = \arctan(\frac{e_{1,i}}{e_{2,i}}) \forall i = 1, \dots, m$. The increasing order of these phases $\theta_{(1)} \prec \theta_{(2)} \prec \cdots \prec \theta_{(m)}$ induces a circular order $\mathbf{o}$ on data collection, see panels (d), (e) and (f) in Fig K.

Each circular order represents $2m$ sampling time configurations depending on the choice of starting point and the direction. To make this choice, CIRCUST relies on three standard assumptions: (1) The peak phase of *ARNTL* is set at π which induces a starting

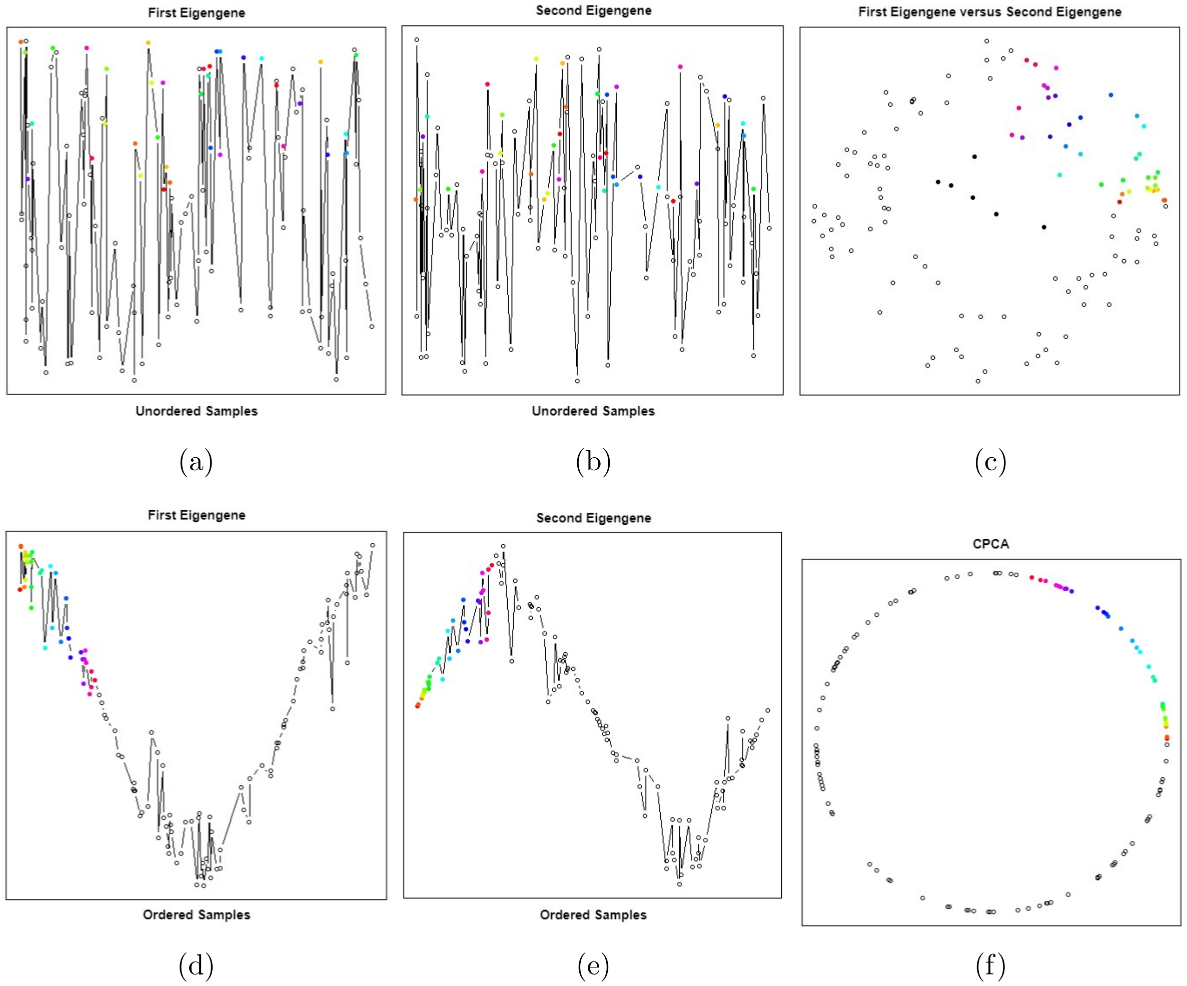

Figure K: CPCA performance illustration. (a): First eigengene from the unordered expression matrix. (b): Second eigengene from the unordered expression matrix. (c): (a) versus (b) mapping. Black dots are outliers samples to be deleted, see Section 3.3 (d): First eigengene ordered regarding the increased order of the angular values given in $\boldsymbol{\theta}$. (e): Second eigengene ordered regarding the increased order of the angular values given in $\boldsymbol{\theta}$. (f) Eigengenes' projection onto the unit circle regarding the increased order of the angular values given in $\boldsymbol{\theta}$. Colors illustrate the order among the samples with the lower angular values in $\boldsymbol{\theta}$.

point and facilitates comparisons. Moreover, as it is well-known that *ARNTL* peaks in anticipation of the inactive period in mammals [6], then in this work $[0, \pi)$ and $[\pi, 2\pi)$ represent inferred light and dark periods, respectively; (2) *DBP* peaks after ROR-phased genes (*ARNTL, NPAS, CLOCK*) [7, 8, 9]; (3) Peaks phases for the most of the core clock genes occur while the active period $[0, \pi)$ [10, 11]. These two latter assumptions define clockwise or counterclockwise direction.

In CIRCUST$_{prior}$ the assumption (2) can be refined, in terms of peak phases' order restrictions, incorporating additional knowledge regarding the molecular clock network of the species or experiment.

3.3 CPCA Outliers sample detection

The role of CPCA in this work is twofold. In addition to providing a solution for the temporal order estimation problem, it can be used, in conjunction with the FMM predictions, as an outlier identification that facilitates the detection of different rhythmicity disruptions based on two different criteria.

Consider the mapping of the two eigengenes computed on the $[\boldsymbol{N}]$ from the 12 core clock genes. The observation pairs samples at a neighbourhood of the origin $(0, 0)$ disrupt the underlying circular structure of the data, see the inner black dots panel (c) of Fig K. Let L_{E_i} denote the distance in absolute value between $(0, 0)$ and the pairs $(E_{1,i}, E_{2,i})$, for $i = 1, \ldots, m$. This first radial distance criterion addresses outlier sample detection likely related to circadian clock misalignments reflecting individual variability. Next, second criterion, based on FMM residuals is designed to exclude common outlier samples across genes, such as those derived from devices' measurement errors, among other factors. The FMM adequacy is assessed based on the standardized residuals $(r_i, i = 1, \ldots, n)$ of the 12 core clock genes. Finally, paired samples violating $L_{E_i} < 0.1$ or $r_i > 3$ are declared as outliers and deleted from $[\boldsymbol{N}]$.

3.4 Seed gene selection and circular ordering

This section describes the robustness of the circular ordering against changes on the 12-seed gene list provided. To investigate this issue, for each tissue we have recomputed the orderings changing the seed genes selection leaving out each time one seed gene. It is worth noting that *ARNTL, DBP*, and *CRY1* were retained in the analysis due to their crucial role as synchronization cues. To compare the original ordering (using all 12 seed genes) with the recomputed orderings obtained across tissues, we employed the circular correlation coefficient. The median (and standard deviation) value of the circular correlation was 0.969 (0.016). As an example, Fig L illustrates the correlation between the original ordering and the recomputed orderings for adipose-subcutaneous tissue, with each of the 9 possible seed genes. For all the cases, a simple visual inspection shows a high concordance between the orders.

3.5 R^2-based goodness of fit criteria

Given a rhythmicity model (ORI, FMM, Cosinor), this work employs a R^2-based goodness of fit criteria to assess rhythmicity following the lines given in [2, 12]. This measure is defined as follows:

$$R^2_{Model}(\boldsymbol{X}^g) = \frac{\sum_{i=1}^{m}(X_i^g - \hat{X}_i^g)^2}{\sum_{i=1}^{m}(X_i^g - \overline{X}^g)^2}, \tag{1}$$

where $\boldsymbol{X}^g = (X_1^g, \ldots, X_m^g)'$ denotes the gene expression of gene g, $\hat{\boldsymbol{X}}^g$ is the predicted expression pattern from the rhythmicity model and $\overline{X}^g$ denotes $\boldsymbol{X}^g$ average expression value.

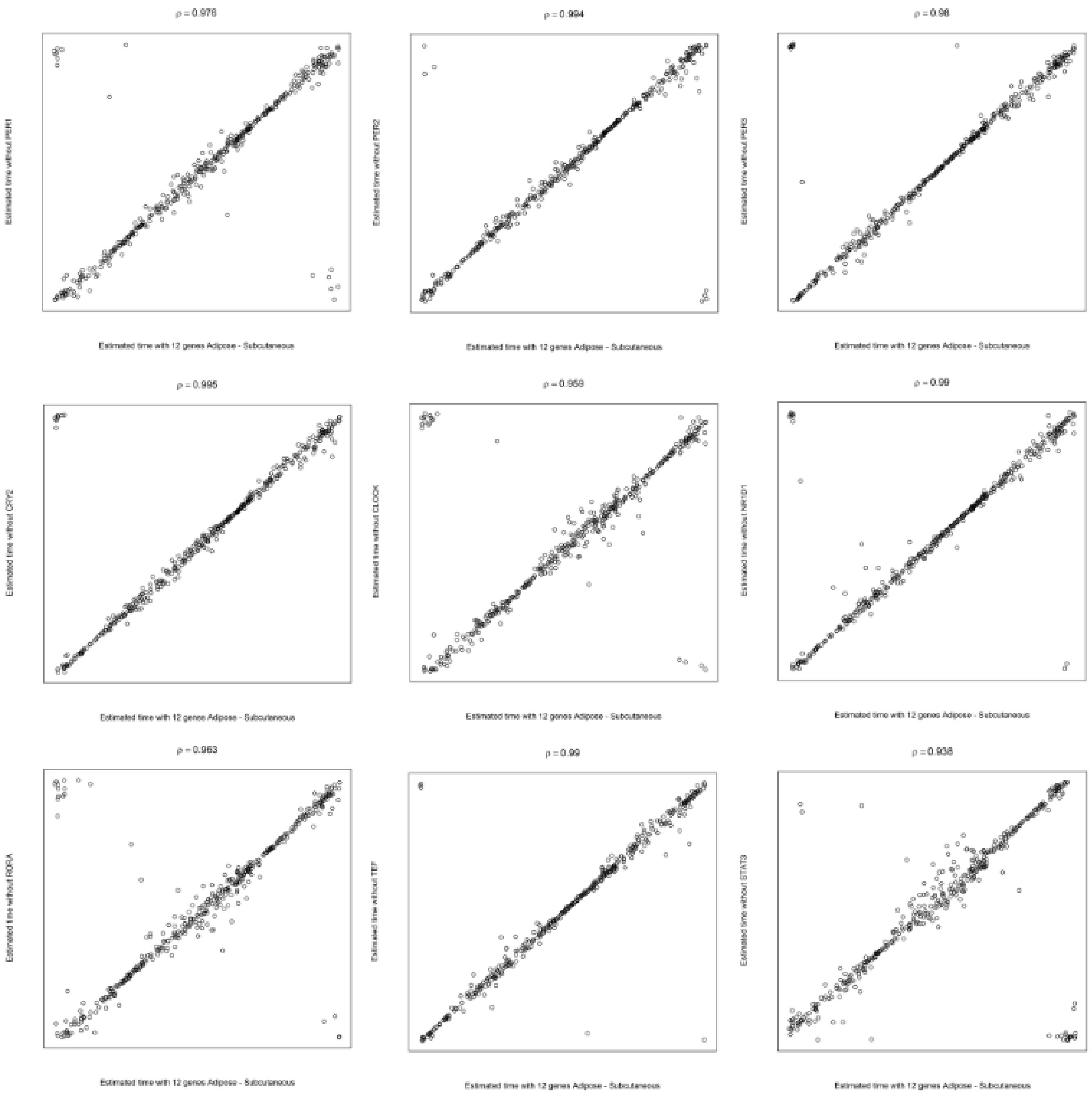

Figure L: Original *versus* recomputed ordering in Adipose-Subcutaneous tissue for nine seed genes. X-axis: original ordering. Y-axis: recomputed orderings leaving out one seed gene each time. Circular correlation is given at the top. The points at the bottom right and top left are closely positioned as both axes span from 0 to 2π.

R^2-based goodness of fit measure is employed in this work instead of p-values. The rationale behind this is that p-values are not a good measure to quantify rhythmicity in this dataset as they highly depend on the sample size which drastically varies from one tissue to another. In any case, FMM rhythmicity tests can be defined using the likelihood ratio for testing H_0: Flat against H_1 : FMM or even H_0: Cosinor against H_1: FMM. One may refer to [2] for details.

4 Supplementary results: Simulation

The aim of the following simulation study is to assess the performance of CPCA methodology, at the core of the CIRCUST algorithm, solving temporal order estimation when faced with symmetric and asymmetric patterns, non-rhythmic confounds, equally and non-equally sampling distributions, and several noise scenarios. The FMM model's adequacy in the analysis of oscillatory patterns is also evinced.

Simulated data were generated from five signals that represent real gene expression patterns: two rhythmic and symmetric (called *Symm1,Symm2*), two rhythmic and non-symmetric (called *Asymm1,Asymm2*) and one non-rhythmic or flat signal (called *Flat*). The four rhythmic signals were simulated from the FMM model, Table E details the parameter configuration used. An illustration of these signals is shown in the first row of Figs M and N. Corresponding to each signal, we simulate data $\boldsymbol{X} = (X_1, \ldots, X_m)'$ for $m = 25, 50, 100$ using the simulated data set equation $\boldsymbol{X} \sim N_m(\boldsymbol{\mu}, \sigma^2\boldsymbol{I})$, where $\sigma^2 = 0.025, 0.05$. The values of $\boldsymbol{\mu}$ are chosen so as to represent the five different signals considered. Moreover, we considered two scenarios for sampling times distributions: equally-spaced and non-equally, based on uniform distribution, in $[0, 2\pi)$.

For each combination of noise, sampling size and time distribution, a dataset with 10000 patterns is generated, 40% corresponding to rhythmic patterns (a thousand for each of the four rhythmic signals) and the rest to non-rhythmic patterns, mimicking gene studies. Then, data are randomly unordered, and CPCA temporal order algorithm is conducted. Finally, FMM model is fitted to the reordering patterns. A representative illustration of the performance of this algorithm is given in Figs M and N for the case of $\sigma^2 = 0.05$.

The numerical results in Table F show the differences between the true and estimated time peaks (t_U) from the recovered patterns in terms of the MSE. Differences generally decrease according to sample size and noise, being slightly lower for the case of equally-spaced sampling times. Among the patterns considered, *Asymm2* is the pattern that presents worse results, especially for non-equally spaced data. Nevertheless, the differences significantly reduce for larger samples. According to the graphical and numerical results, differences seem reasonable for all the scenarios considered. MSE computation of t_U relies on the cosine distance as time distribution is an angular variable. Data were synchronized regarding *Symm1*.

	Symm1	*Asymm1*	*Simm2*	*Asymm2*
α	0	0	$\pi/2$	π
β	$3\pi/2$	$3\pi/2$	$3\pi/2$	$3\pi/2$
ω	0.9	0.1	0.9	0.1

Table E: FMM parameter configuration for rhythmic signals used in simulation with $M = 0$ and $A = 1$.

		Equally spaced				Non-equally-spaced			
σ^2	n	*Symm1*	*Asymm2*	*Symm2*	*Asymm2*	*Symm1*	*Asymm2*	*Symm2*	*Asymm2*
0.025	n=25	0.000	0.039	0.002	0.018	0.000	0.066	0.032	0.377
	n=50	0.000	0.025	0.001	0.005	0.000	0.005	0.017	0.175
	n=100	0.000	0.011	0.001	0.002	0.000	0.003	0.001	0.007
0.05	n=25	0.000	0.041	0.003	0.048	0.000	0.043	0.008	0.141
	n=50	0.000	0.024	0.003	0.031	0.000	0.012	0.010	0.006
	n=100	0.000	0.008	0.002	0.007	0.000	0.005	0.007	0.022

Table F: MSE for t_U on the simulated dataset across different scenarios of size, noise and sampling frequency distribution.

5 CIRCUST comparison against CYCLOPS and CHIRAL

This section compares the main aspects regarding three relevant methodologies for solving the temporal in the literature used in this paper.

5.1 CIRCUST against CYCLOPS

This first comparison specifically focuses on the methodological differences between CIRCUST and CYCLOPS.

The orderings provided by both methods are based on the arctan transformation of two variables. This transformation, discussed in [13, 5], provides the temporal position of samples in the reconstructed order. However, there are differences in the genes from which these two variables are generated and in the criteria to compute them. On the one hand, CYCLOPS combines the prior knowledge in mice to select a set of homologous genes with the use of a circular node autoencoder. In particular, CYCLOPS constrains the size of the "bottleneck layer" of this autoencoder network to encode the data in a reduced number of dimensions (eigengenes). CYCLOPS weight and combines eigengenes to identify the closed curve (with arctan) that best represents the underlying periodic process.

On the other hand, CIRCUST computes the two eigengenes using PCA from a set of 12 seed rhythmic genes from which are selected to define a preliminary order using arctan transformation (CPCA). This order serves to set the tissue-specific set of TOP rhythmic genes. This process, based on CPCA, is repeated using as input random selection of these TOP genes to determine the final orders of each tissue. The preliminary order step also incorporates an outlier detection step. More details regarding these steps are given in Section 2.2 of the main and Section 3.2 of the Supporting Information.

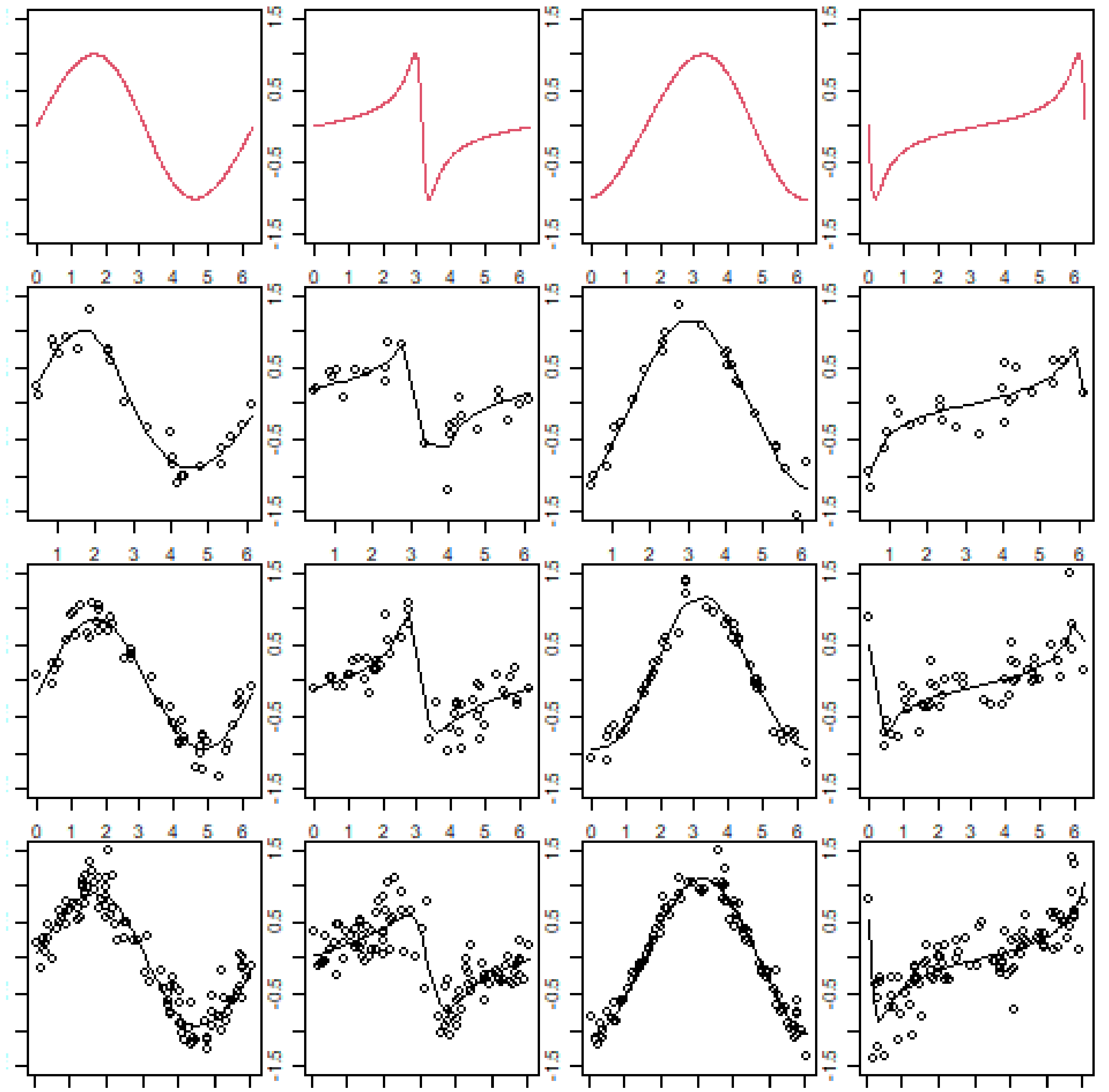

Figure M: True and reordered selected patterns when times are equally-spaced distributed and $\sigma^2 = 0.05$. First row displays in red the four true rhythmic signals generated from FMM. Second, third and fourth row display in black the reordered patterns after solving temporal order estimation for $n = 25, 50, 100$, respectively. Superposed curves correspond to FMM fitting.

5.2 CIRCUST against CHIRAL

The differences between the two procedures are numerous and refer to the objective, the initial biological assumptions, the preprocessing, and the methodology, which consequently together affect the results. Below we will point out the most relevant ones.

A primary difference between our paper and [14] is the objective of the study. Both works incorporate specific algorithms to solve the temporal order estimation problem, named CIRCUST and CHIRAL. However, the role that each algorithm plays is different.

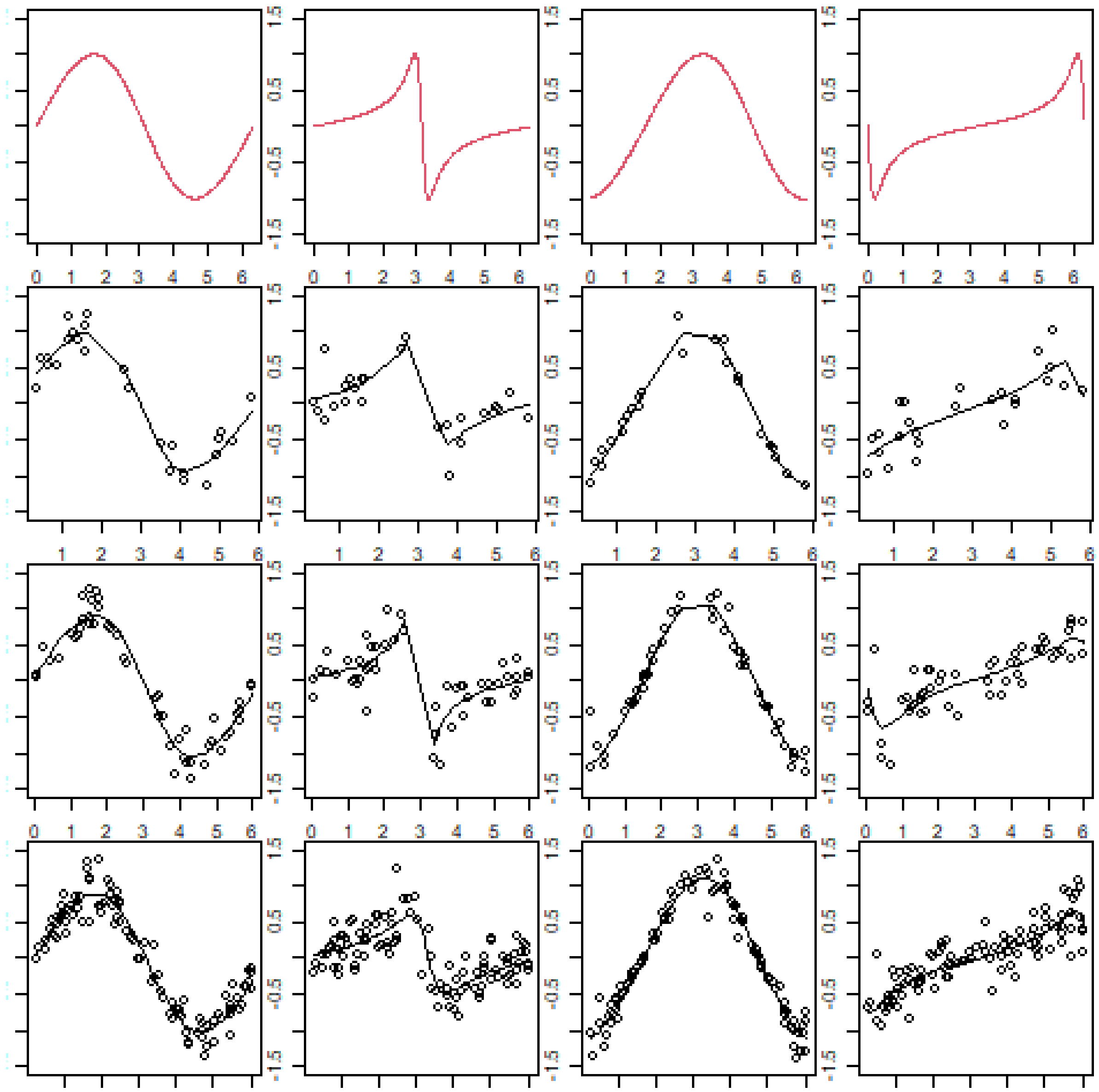

Figure N: True and reordered selected patterns when times are non-equally-spaced distributed and $\sigma^2 = 0.05$. First row displays in red the four true rhythmic signals generated from FMM. Second, third and fourth row display in black the reordered patterns after solving temporal order estimation for $m = 25, 50, 100$, respectively. Superposed curves correspond to FMM fitting.

For a given tissue, the main objective of our work is the identification of rhythmic genes and the estimation of their peaks whereas in [14] the phases are the main objective.

Secondly, the selection of samples and tissues, the reference genes, and the preprocessing are also different. Our study analyses 34 GTEx tissues and includes an outlier sample detection step, while [14] use the RIN index that results in a more reduction of the sample sizes compare to those in CIRCUST, but it analyses 46 tissues. The smaller list of tissues analyzed in our paper is due to the deletion of brain tissues and cell lines

which has been done as they may induce different molecular networks. Regarding the seed gene selection, both methods consider initially a set of 12 genes, sharing 8 of them. However, CIRCUST makes a secondary tissue-specific selection. Finally, the differences in the preprocessing stage are the normalization method and the covariate adjustment that is only done in CHIRAL. The covariate adjustment could be easily incorporated in CIRCUST, however, it deserves an in-depth analysis.

Thirdly, a main difference, in our opinion, is the biological assumption regarding the invariance or uniqueness of circadian phases across tissues for a given subject, which apparently only affects the case of the analysis of several tissues. In other words, CHIRAL assumes the invariance while CIRCUST assumes tissue-specific phases. Following the notation given in [14], we use the terms DIP (Donor Internal Phase) and TIP (Tissue Inferred Phase) to estimators of the unique and tissue-specific phases, respectively. From a biological point of view this assumption is not well-established in the literature [15, 16]. We have analyzed the invariance assumption in CIRCUST by estimating the DIPs as the TIPs median across tissues for each subject. In terms of the rhythmicity measure, we have found that under the invariance assumption, the R^2_{FMM} values are lower for the ∼95% of the seed genes being the R^2_{FMM} reduction of 54.8%, on average.

This study would be extended in the future to look for more evidence related to this important question. Specifically to establish conditions and/or tissues which guarantee invariant biological times.

Fourthly, considering the phase estimation problem of a single tissue CHIRAL considers a harmonic Gaussian regression model while CIRCUST uses the two principal components from a CPCA. Despite the difference in formulation, the estimation problem is equivalent [17], but there are differences in the estimation algorithm: CHIRAL uses Bayesian inference, and the EM algorithm and CIRCUST directly solves a quadratic optimization problem.

Fifthly, for the analysis of rhythmicity and peak estimation CHIRAL employs the Cosinor model and the p-values, while CIRCUST uses a less restrictive models, the FMM for peak estimation and the R^2_{ORI} and R^2_{FMM} values as rhythmicity measures.

Sixthly, the validation task in both papers is approached differently being more exhaustive in our study. CHIRAL has been validated only for single tissue performance: against the true phases in 12 mouse tissues and against CYCLOPS on a human biopsy dataset from muscle-skeletal. On the other hand, CIRCUST performance has been compared to CYCLOPS on human biopsy samples from skin and to CHIRAL on human biopsy data from muscle-skeletal, the same used in [18]. Moreover, CIRCUST performance has been validated with human autopsy data with reliable TODs, and with data from 47 baboon tissues where the sampling times are known.

Finally, these differences are reflected in differences in the resulting expression patterns across tissues and genes, specifically the peak estimator's distribution is more dispersed with CIRCUST than does with CHIRAL, and CIRCUST identifies more rhythmic genes

and provides higher R^2 values when the TIPs are used.

6 List of acronyms

ASC: Ascending Colon
BA: Brodmann's Area
CPCA: Circular Principal Component Analysis
DIP: Donor Internal Phase
FMM: Frequency Modulated Möbius
GTEx: Genotype-Tissue Expression
MAD: Median Absolute Deviance
ORI: Order Restricted inference
PAN: Pancreas
SKI: Skin
TIP: Tissue Inferred Phase
TOD: Time Of Death
WAM: White Adipose Mesenteric

References

[1] Conway, J. R., Lex, A. & Gehlenborg, N. Upsetr: an r package for the visualization of intersecting sets and their properties. *Bioinformatics* **33**, 2938–2940 (2017).

[2] Rueda, C., Larriba, Y. & Peddada, S. Frequency modulated möbius model accurately predicts rhythmic signals in biological and physical sciences. *Scientific Reports* **9**, 18701 (2019).

[3] Fernández, I. *et al.* Fmm: Rhythmic patterns modeling by fmm models. *R package version 0.3. 0* (2021).

[4] Alter, O., Brown, P. O. & Botstein, D. Singular value decomposition for genome-wide expression data processing and modeling. *Proceedings of the National Academy of Sciences* **97**, 10101–10106 (2000).

[5] Scholz, M. Analysing periodic phenomena by circular pca. In *Proceedings of the Conference on Bioinformatics Research and Development*, vol. 4414, 38–47 (2007).

[6] Ruben, M. D. *et al.* A database of tissue-specific rhythmically expressed human genes has potential applications in circadian medicine. *Science Translational Medicine* **10** (2018).

[7] Wu, G. *et al.* Population-level rhythms in human skin with implications for circadian medicine. *Proceedings of the National Academy of Sciences* **115**, 12313–12318 (2018).

[8] Pett, J. P., Kondoff, M., Bordyugov, G., Kramer, A. & Herzel, H. Co-existing feedback loops generate tissue-specific circadian rhythms. *Life science alliance* **1** (2018).

[9] Mavroudis, P., DuBois, D., Almon, R. & Jusko, W. Modeling circadian variability of core-clock and clock-controlled genes in four tissues of the rat. *PloS one* **13**, e0197534. (2018).

[10] Korencic, A. *et al.* Timing of circadian genes in mammalian tissues. *Scientific Reports* **4** (2014).

[11] Mure, L. S. *et al.* Diurnal transcriptome atlas of a primate across major neural and peripheral tissues. *Science* **359** (2018).

[12] Larriba, Y., Rueda, C., Fernández, M. & Peddada, S. Order restricted inference in chronobiology. *Statistics in Medicine* **39**, 265–278 (2020).

[13] Kirby, M. J. & Miranda, R. Circular nodes in neural networks. *Neural Computation* **8**, 390–402 (1996).

[14] Talamanca, L., Gobet, C. & Naef, F. Sex-dimorphic and age-dependent organization of 24-hour gene expression rhythms in humans. *Science* **379**, 478–483 (2023).

[15] Zhu, Y., Wang, L., Yin, Y. & Yang, E. Systematic analysis of gene expression patterns associated with postmortem interval in human tissues. *Scientific reports* **7**, 5435 (2017).

[16] Ferreira, P. G. *et al.* The effects of death and post-mortem cold ischemia on human tissue transcriptomes. *Nature communications* **9**, 490 (2018).

[17] Tipping, M. E. & Bishop, C. M. Probabilistic principal component analysis. *Journal of the Royal Statistical Society Series B: Statistical Methodology* **61**, 611–622 (1999).

[18] Talamanca, L. Statistical physics of periodic biological processes. Tech. Rep., EPFL (2023).

Fig Legends

- Fig A: Core clock gene expression patterns from Skin sun-exposed (Lower leg) from GTEx dataset. Left: gene expressions as a function of TOD times. Right: gene expressions as a function of CIRCUST estimated times ($[0, 2\pi)$).

- Fig B: Outline of the CIRCUST methodology.

- Fig C: Circular-circular regression model for the real clock times (X-axis) and CIRCUST$_{prior}$ estimated times (Y-axis) across the 47 baboons' tissues selected. Horizontal axis: sampling real clock times along 24-h (ZT0,ZT2,...,ZT22). Vertical axis: CIRCUST estimated times in $[0, 2\pi)$. Time 0-h is the same as 24-h and the phase 0 is the same as 2π. The diagonal line observed for most of the tissues is used as a marker of the coherence between the orders. Colors match with the 12 functional organs groups considered in GSE98965. See Table A for tissue names.

- Fig D: Expression of selected clock genes *NFIL3*, *NPAS2*, *NR1D2* and *HLF* in White Adipose Mesenteric (WAM) tissue from baboons (GSE98965). Top panels: expressions as function of known times ZT0, ZT2,...,ZT22. Bottom panels: expressions as function of CIRCUST estimated times. FMM predictions are shown as blue solid lines. Cosinor predictions are shown as red solid lines.

- Fig E: Expression of selected clock genes *NFIL3*, *NPAS2*, *NR1D2* and *HLF* in Ascending Colon (ASC) tissue from baboons (GSE98965). Top panels: expressions as function of known times ZT0, ZT2,...,ZT22. Bottom panels: expressions as function of CIRCUST estimated times. FMM predictions are shown as blue solid lines. Cosinor predictions are shown as red solid lines.

- Fig F: Expression of selected clock genes *NFIL3*, *NPAS2*, *NR1D2* and *HLF* in Skin (SKI) tissue from baboons (GSE98965). Top panels: expressions as function of known times ZT0, ZT2,...,ZT22. Bottom panels: expressions as function of CIRCUST estimated times. FMM predictions are shown as blue solid lines. Cosinor predictions are shown as red solid lines.

- Fig G: Gene expression of seed genes in White Adipose Mesenteric (WAM) tissue from baboons (GSE98965) after discarding outliers. In Fig D, ZT0 may seem to be outlier looking at *HLF* and *NR1D2*. CIRCUST does not delete ZT0 because it is not outlier for the seed genes *PER1, PER2, CRY1, CRY2* or *ARNTL*. FMM predictions are shown as solid lines.

- Fig H: Number of TOP rhythmic genes across the GTEx. Tissues are shown in decreasing the number of TOP rhythmic genes.

- Fig I: UpSetR [1] technique to visualize TOP rhythmic gene intersections across the 34 tissues. Dots denote single tissue intersections. Bars match tissues with common TOP rhythmic genes. For better illustration, only the eight tissues with the higher number of TOP genes have been included in the analysis.

- Fig J: FMM robustness against outliers samples. Simulated data from **FMM** package [3] covering different patterns and with 50 (left), 100 (middle) and 200 (right) observations.

- Fig K: CPCA performance illustration. (a): First eigengene from the unordered expression matrix. (b): Second eigengene from the unordered expression matrix. (c): (a) versus (b) mapping. Black dots are outliers samples to be deleted, see Section 3.3 (d): First eigengene ordered regarding the increased order of the angular values given in $\boldsymbol{\theta}$. (e): Second eigengene ordered regarding the increased order of the angular values given in $\boldsymbol{\theta}$. (f) Eigengenes' projection onto the unit circle regarding the increased order of the angular values given in $\boldsymbol{\theta}$. Colors illustrate the order among the samples with the lower angular values in $\boldsymbol{\theta}$.

- Fig L: Original *versus* recomputed ordering in Adipose-Subcutaneous tissue for nine seed genes. X-axis: original ordering. Y-axis: recomputed orderings leaving out one seed gene each time. Circular correlation is given at the top. The points at the bottom right and top left are closely positioned as both axes span from 0 to 2π.

- Fig M: True and reordered selected patterns when times are equally-spaced distributed and $\sigma^2 = 0.05$. First row displays in red the four true rhythmic signals generated from FMM. Second, third and fourth row display in black the reordered patterns after solving temporal order estimation for $m = 25, 50, 100$, respectively. Superposed curves correspond to FMM fitting.

- Fig N: True and reordered selected patterns when times are equally-spaced distributed and $\sigma^2 = 0.05$. First row displays in red the four true rhythmic signals generated from FMM. Second, third and fourth row display in black the reordered patterns after solving temporal order estimation for $m = 25, 50, 100$, respectively. Superposed curves correspond to FMM fitting.

Table Legend

- Table A: Baboons tissue characterization. First column: Tissue abbreviation. Second column: Tissue name. Third column: R^2_{Ave}, average rhythmicity measure R^2_{FMM} for the 12 core clock genes as a function of estimated times. Fourth column: ρ goodness of fit measure fit measure for circular-circular regression between the real times (ZT0,ZT2,...,ZT22) and the CIRCUST estimated times ($[0, 2\pi)$). Tissues

are restricted to those with $R^2_{Ave} > 0.7$ to guarantee the consistency of the results. Lines separate functional groups.

- Table B: GTEx donor distribution by sex, age, and cause of death. Death was classified as follows. Fast: death due to accident, blunt force trauma, or suicide; Intermediate: patients who were ill but death was unexpected; Slow: death after a long illness; Sudden-Natural: fast death of natural causes, sudden unexpected deaths; Ventilator: all cases on a ventilator immediately before death.

- Table C: R^2_{FMM} for the time course expression of the 12 core clock genes as a function of CIRCUST times across the 34 GTEx tissues analyzed. Only the peaks of the core clock genes with $R^2_{FMM} > 0.3$ are shown in Fig 9.

- Table D: Estimated t_U for the time course expression of the 12 core clock genes as a function of CIRCUST times across the 34 GTEx tissues analyzed. Only the peaks of the core clock genes with $R^2_{FMM} > 0.3$ are shown in Fig 9.

- Table E: FMM parameter configuration for rhythmic signals used in simulation with $M = 0$ and $A = 1$.

- Table F: MSE for t_U on the simulated dataset across different scenarios of size, noise and sampling frequency distribution.
